# Supplementary material for: Yiai Fuzheng decoction inhibits triple-negative breast cancer by remodeling the immune microenvironment
Source: Front Immunol. 2025 Sep 30;16:1615631. doi: 10.3389/fimmu.2025.1615631 (PMC12518410; doi:10.3389/fimmu.2025.1615631)
Supplement: Supplementary file 2 [file DataSheet1.zip › Supplementary File 2/Treatment/C_vs_H/07_Pathway_Analysis/Pathway_Analysis/Sig_Pathways_ID/Purine metabolism.pdf]

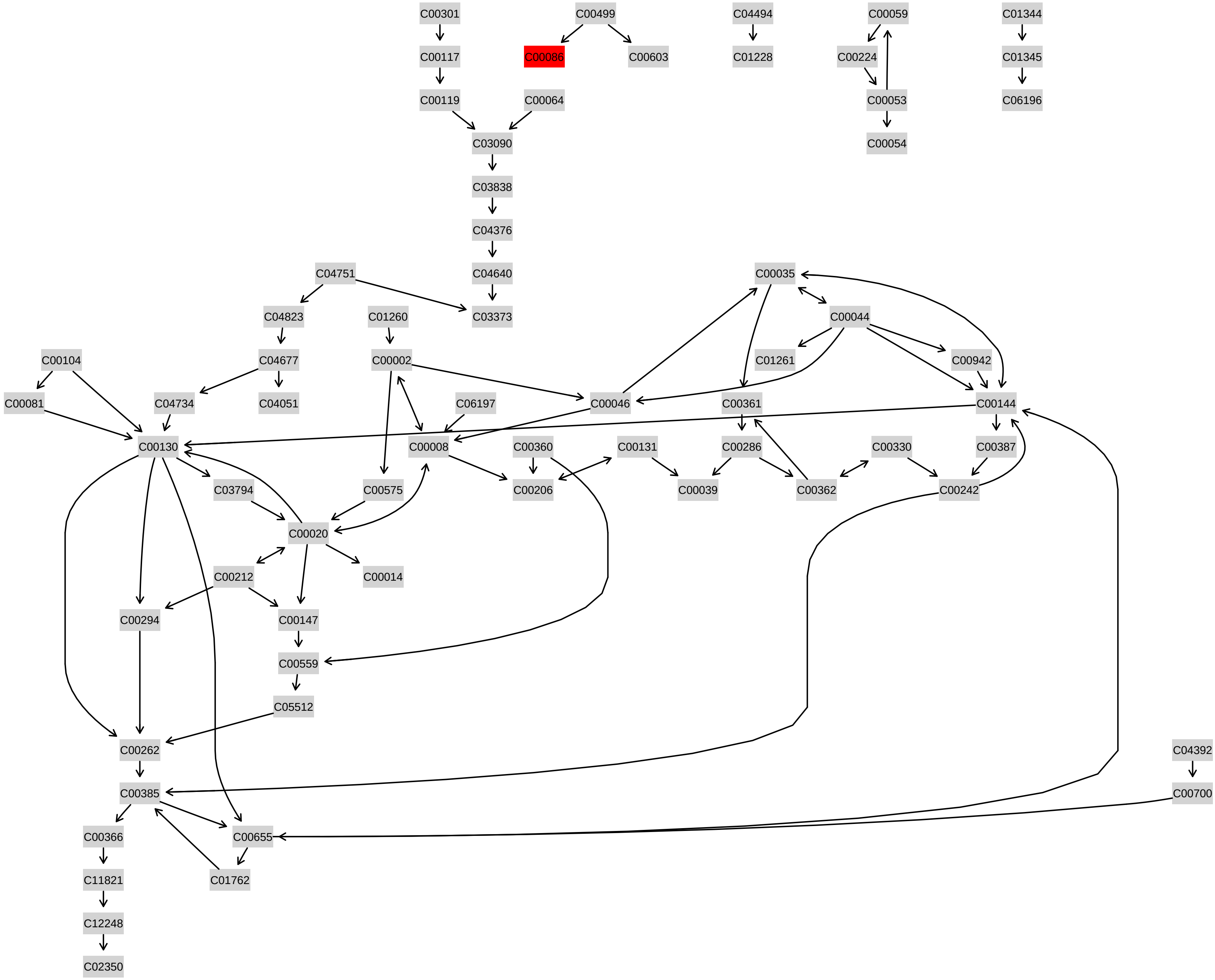
C00301
C00499
C04494
C00059
C01344
C00117
C00086
C00603
C01228
C00224
C01345
C00119
C00064
C00053
C06196
C03090
C00054
C03838
C04376
C04751
C04640
C00035
C04823
C01260
C03373
C00044
C00104
C04677
C00002
C01261
C00942
C00081
C04734
C04051
C06197
C00046
C00361
C00144
C00130
C00008
C00360
C00131
C00286
C00330
C00387
C03794
C00575
C00206
C00039
C00362
C00242
C00020
C00212
C00014
C00294
C00147
C00559
C05512
C00262
C04392
C00385
C00700
C00366
C00655
C11821
C01762
C12248
C02350
